# Parametric Alignment of Drosophila Genomes

**DOI:** 10.1371/journal.pcbi.0020073

**Published:** 2006-06-23

**Authors:** Colin N Dewey, Peter M Huggins, Kevin Woods, Bernd Sturmfels, Lior Pachter

**Affiliations:** 1 Department of Electrical Engineering and Computer Sciences, University of California Berkeley, Berkeley, California, United States of America; 2 Department of Mathematics, University of California Berkeley, Berkeley, California, United States of America; University of California Davis, United States of America

## Abstract

The classic algorithms of Needleman–Wunsch and Smith–Waterman find a maximum a posteriori probability alignment for a pair hidden Markov model (PHMM). To process large genomes that have undergone complex genome rearrangements, almost all existing whole genome alignment methods apply fast heuristics to divide genomes into small pieces that are suitable for Needleman–Wunsch alignment. In these alignment methods, it is standard practice to fix the parameters and to produce a single alignment for subsequent analysis by biologists. As the number of alignment programs applied on a whole genome scale continues to increase, so does the disagreement in their results. The alignments produced by different programs vary greatly, especially in non-coding regions of eukaryotic genomes where the biologically correct alignment is hard to find. Parametric alignment is one possible remedy. This methodology resolves the issue of robustness to changes in parameters by finding all optimal alignments for all possible parameters in a PHMM. Our main result is the construction of a whole genome parametric alignment of Drosophila melanogaster and Drosophila pseudoobscura. This alignment draws on existing heuristics for dividing whole genomes into small pieces for alignment, and it relies on advances we have made in computing convex polytopes that allow us to parametrically align non-coding regions using biologically realistic models. We demonstrate the utility of our parametric alignment for biological inference by showing that cis-regulatory elements are more conserved between Drosophila melanogaster and Drosophila pseudoobscura than previously thought. We also show how whole genome parametric alignment can be used to quantitatively assess the dependence of branch length estimates on alignment parameters.

## Synopsis

Dewey and colleagues describe a parametric alignment of the genomes of Drosophila melanogaster and Drosophila pseudoobscura. The parametric alignment consists of all optimal alignments of the two *Drosophila* genomes for all choices of parameters for some widely used scoring schemes. Computation and analysis of the parametric alignment requires the integration of ideas from mathematics, algorithms, and biology. Mathematically, the parametric analysis rests on the geometric principle of convexity. In particular, the alignment polytope, which organizes the alignments according to the optimal alignments, is introduced and described. Algorithmically, efficient procedures are developed for computing alignment polytopes on a large scale and for models with more parameters than had previously been practical. Biologically, the utility of parametric analysis is demonstrated by showing that the degree of conservation between cis-regulatory elements in Drosophila melanogaster and Drosophila pseudoobscura is higher than previously thought, and by assessing the dependence of branch length estimates on alignment parameters.

## Introduction

Needleman–Wunsch pairwise sequence alignment [1] is known to be sensitive to parameter choices. To illustrate the problem, consider the eighth intron of the *Drosophila melanogaster CG9935-RA* gene (as annotated by FlyBase [2]) located on chr4:660,462–660,522 (April 2004 BDGP release 4). This intron, which is 61 base pairs long, has a 60 base pair–ortholog in Drosophila pseudoobscura. The ortholog is located at Contig8094_Contig5509:4,876–4,935 in the August 2003 freeze 1 assembly, as produced by the Baylor Genome Sequencing Center.

Using the basic 3-parameter scoring scheme (match *M,* mismatch *X,* and space penalty *S*), these two orthologous introns have the following optimal alignment when the parameters are set to *M = 5, X = −5, S = −5:*


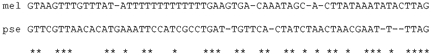


However, if we change the parameters to *M =* 5, *X =* −6, and *S =* −4, then the following alignment is optimal:


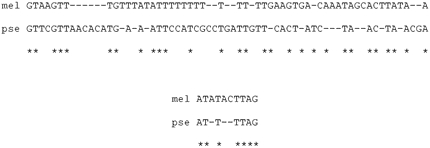


Note that a relatively small change in the parameters produces a very different alignment of the introns. This problem is exacerbated with more complex scoring schemes, and is a central issue with whole genome alignments produced by programs such as MAVID [3] or BLASTZ/MULTIZ [4]. Indeed, although whole genome alignment systems use many heuristics for rapidly identifying alignable regions and subsequently aligning them, they all rely on the Needleman–Wunsch algorithm at some level. Dependence on parameters becomes an even more crucial issue in the multiple alignment of more than two sequences.

Parametric alignment was introduced by Waterman, Eggert, and Lander [5] and further developed by Gusfield et al. [6,7] and Fernandez-Baca et al. [8] as an approach for overcoming the difficulties in selecting parameters for Needleman–Wunsch alignment. See [9] for a review and [10,11] for an algebraic perspective. Parametric alignment amounts to partitioning the space of parameters into regions. Parameters in the same region lead to the same optimal alignments. Enumerating all regions is a non-trivial problem of computational geometry. We solve this problem on a whole genome scale for up to five free parameters.

Our approach to parametric alignment rests on the idea that the score of an alignment is specified by a short list of numbers derived from the alignment. For instance, given the standard three-parameter scoring scheme, we summarize each alignment by the number *m* of matches, the number *x* of mismatches, and the number *s* of spaces in the alignment. The triple (*m*,*x*,*s*) is called the *alignment summary*. As an example, consider the above pair of orthologous *Drosophila* introns. The first (shorter) alignment has the alignment summary (33,23,9) while the second (longer) alignment has the alignment summary (36,10,29).

Remarkably, even though the number of all alignments of two sequences is very large, the number of alignment summaries that arise from Needleman–Wunsch alignment is very small. Specifically, in the example above, where the two sequences have lengths 61 and 60, the total number of alignments is 1,511,912,317,060,120,757,519,610,968,109,962,170,434,175,129 ≈ 1.5×10^46^.

There are only 13 alignment summaries that have the highest score for some choice of parameters *M*,*X*,*S*. For biologically reasonable choices, i.e., when we require *M* > *X* and 2*S* < *X,* only six of the 13 summaries are optimal. These six summaries account for a total of 8,362 optimal alignments (Table 1).

**Table 1:**
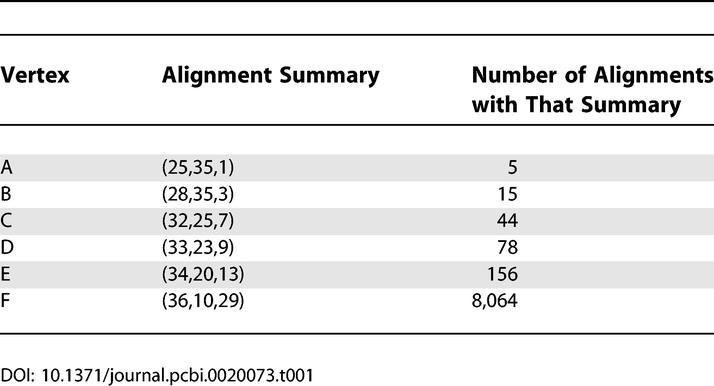
The 8,362 Optimal Alignments for Two *Drosophila* Intron Sequences

Note that the basic model discussed above has only *d* = 2 free parameters, because for a pair of sequences of lengths *l*,*l* all the summaries (*m*,*x*,*s*) satisfy


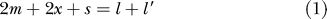


This relation holds with *l + l′* for the six summaries in Table 1. Figure 1 shows the alignment polygon, as defined in the section “Alignment polytopes,” in the coordinates (*x*,*s*).

**Figure 1:**
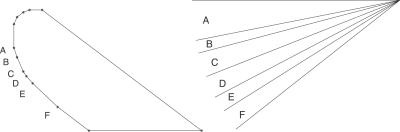
The Alignment Polygon for Our Two Introns Is Shown on the Left For each of the alignment summaries *A,B, . . . F* in Table 1, the corresponding cone in the alignment fan is shown on the right. If the parameters (*S,X*) stay inside a particular cone, every optimal alignment has the same alignment summary.

In general, for two DNA sequences of lengths *l* and *l′,* the number of optimal alignment summaries is bounded from above by a polynomial in *l* and *l′* of degree *d*(*d* − 1)/(*d* + 1), where *d* is the number of *free parameters* in the model [9,10]. For *d* = 2, this degree is 0.667, and so the number of optimal alignment summaries has *sublinear growth* relative to the sequence lengths. Even for *d* = 5, the growth exponent *d*(*d* − 1)/(*d* + 1) is only 3.333. This means that *all* optimal alignment summaries can be computed on a large scale for models with few parameters.

The growth exponent *d*(*d* − 1)/(*d* + 1) was derived by Gusfield et al. [6] for *d* = 2 and by Fernandez-Baca at al. [8] and Pachter-Sturmfels [10] for general *d*. Table 1 can be computed using the software XPARAL [7]. This software works for *d* = 2 and *d* = 3, and it generates a representation of all optimal alignments with respect to all reasonable choices of parameters. Although XPARAL has a convenient graphical interface, it seems that this program has not been widely used by biologists, perhaps because it is not designed for high throughput data analysis and the number of free parameters is restricted to *d* ≤ 3.

In this paper, we demonstrate that parametric sequence alignment can be made practical on the whole-genome scale, and we argue that computing output such as Table 1 can be very useful for comparative genomics applications where reliable alignments are essential. To this end, we introduce a mathematical point of view, based on the organizing principle of *convexity,* which was absent in the earlier studies [5,6,9]. Our advances rely on new algorithms, which are quite different from what is implemented in XPARAL, and which perform well in practice, even if the number *d* of free parameters is greater than three.

Convexity is the organizing principle that reveals the needles in the haystack. In our example, the “haystack” consists of more than 10^46^ alignments, and the “needles” are the 8,362 optimal alignments. The summaries of the optimal alignments are the vertices of the *alignment polytope*. The alignment polytope is the *convex hull* of the summaries of all (exponentially many) alignments. Background on convex hulls and how to compute the alignment polytopes are provided in the section From Genomes to Polytopes (see also [11]). Thus, parametric alignment of two DNA sequences relative to some chosen pair hidden Markov model (PHMM) means constructing the alignment polytope of the two sequences. The dimension of the alignment polytope is *d,* the number of free model parameters. For *d* = 2 (the basic model), the polytope is a convex polygon, as shown in Figure 1 for the pair of introns above.

The basic model is insufficient for genomics applications. More realistic PHMMs for sequence alignment include gap penalties. We consider three such models. The symmetries of the scoring matrices for these models are derived from those of the evolutionary models known as Jukes–Cantor (*d* = 3), Kimura-2 (*d* = 4), and Kimura-3 (*d* = 5). The models are reviewed in the section “Models, alignment summaries, and robustness cones.”

Our contribution is the construction of a whole genome parametric alignment in all four models for D. melanogaster and D. pseudoobscura. Our methods and computational results are described in the next section. Three biological applications are presented in the section From Polytopes to Biology. A discussion follows in the Discussion section.

### From Genomes to Polytopes

Our main computational result is the construction of a whole genome parametric alignment for two *Drosophila* genomes. This result depended on a number of innovations. By adapting existing orthology mapping methods, we were able to divide the genomes into 1,999,817 pairs of reliably orthologous segments, and among these we identified 877,982 pairs for which the alignment is uncertain. We computed the alignment polytopes of dimensions two, three, and four for each of these 877,982 sequence pairs, and of dimension five for a subset of them. The methods are explained in the section “Alignment polytopes.” The vertices of these polytopes represent the optimal alignment summaries and the robustness cones. These concepts are introduced in the section “Models, alignment summaries, and robustness cones.” Computational results are presented in the section “Computational results.”

#### Orthology mapping.

The *orthology mapping problem* for a pair of genomes is to identify all orthologous segments between the two genomes. These orthologous segments, if selected so as not to contain genome rearrangements, can then be globally aligned to each other. This strategy is frequently used for whole genome alignment [12,13], and we adapted it for our parametric alignment computation.

MERCATOR is an orthology mapping program suitable for multiple genomes that was developed by Dewey et al. [14]. We applied this program to the D. melanogaster and D. pseudoobscura genomes to identify pieces for parametric alignment. The MERCATOR strategy for identifying orthologous segments is as follows. Exon annotations in each genome are translated into amino acid sequences and then compared with each other using BLAT [15]. The annotations are based on reference gene sets, and on ab initio predictions (see Materials and Methods). The resulting exon hits are then used to build a graph whose vertices correspond to exons, and with an edge between two exons if there is a good hit. A greedy algorithm is then used to select edges in the graph that correspond to runs of exons that are consistent in order and orientation.

The MERCATOR orthology map for D. melanogaster and D. pseudoobscura has 2,731 segments. However, in order to obtain a map suitable for parametric alignment, further subdivision of the segments was necessary. This subdivision was accomplished by the additional step of identifying and fixing exact matches of length at least 10 bp (see Materials and Methods).

We derived 1,116,792 constraints, which are of four possible types: 1) exact matching non-coding sequences, 2) ungapped high scoring aligned coding sequences, 3) segment pairs between two other constraints where one of the segments has length zero, so the non-trivial segment must be gapped, and 4) single nucleotide mismatches that are squeezed between other constraints.

We then removed all segments where the sequences contained the letter N (which means the actual sequence is uncertain). This process resulted in 877,982 pairs of segments for parametric alignment. The lengths of the D. melanogaster segments range from one to 80,676 base pairs. The median length is 42 bp and the mean length is 99 bp. In all, 90.4% of the D. melanogaster genome and 88.7% of the D. pseudoobscura genome were aligned by our method.

#### Models, alignment summaries, and robustness cones.

For each of the 877,982 pairs of orthologous segments, we constructed all optimal Needleman–Wunsch alignments, with respect to various scoring schemes that are derived from PHMMs. We considered models with two, three, four, and five parameters. See [16] for a review of PHMMs and their relationship to the scoring schemes typically used for aligning DNA sequences. In what follows, we only refer to the scores, which are the logarithms of certain ratios of the parameters of the PHMM. Our four models are specializations of the general 33-parameter model [11] that incorporates mutations, insertions, and deletions of DNA sequences. It is customary to reduce the dimension by assuming that many of the 33 parameters are equal to each other.

The *basic model,* discussed in the Introduction, has three natural parameters, namely, *M* for match, *X* for mismatch, and *S* for space. If the numbers *M, X,* and *S* are fixed, then we seek to maximize *M* · *m* + *X* · *x* + *S* · *s,* where (*m*,*x*,*s*) runs over the summaries of all alignments. In light of Equation 1, this model has only two free parameters and there is no loss of generality in assuming that the match score *M* is zero. From now on we set *M* = 0 and take *X* and *S* as the free parameters. We define the *alignment summary* to be the pair (*x*,*s*).

Following the convention of Pachter and Sturmfels [11], we summarize a scoring scheme with a 5 × 5 matrix *w* whose rows and columns are both indexed by A, C, G, T, and -. The matrix *w* for the basic model is the leftmost matrix in Figure 2, and it corresponds to the Jukes–Cantor model of DNA sequence evolution.

**Figure 2:**
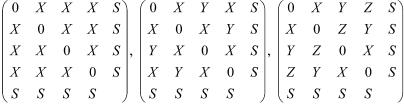
The Jukes–Cantor Matrix, Kimura-2 Matrix, and Kimura-3 Matrix These three matrices correspond to JC69, K80, and K81 in the Felsenstein hierarchy [11] of probabilistic models for DNA sequence evolution.

Our *3d model* is the most commonly used scoring scheme for computing alignments. This model includes the number g of gaps. A *gap* is a complete block of spaces in one of the aligned sequences; it either begins at the start of the sequence or is immediately preceded by a nucleotide, and it either follows the end of the sequence or is succeeded by a nucleotide. The *3d alignment summary* is the triple (*x*,*s*,*g*). The score for a gap, *G,* is known as the *affine gap penalty*. If *X, S,* and *G* are fixed, then the alignment problem is to maximize *X* · *x* + *S* · *s* + *G* · *g* where (*x*,*s*,*g*) runs over all 3d alignment summaries. The parametric version is implemented in XPARAL. Introducing the gap score *G* does not affect the matrix *w,* which is still the leftmost matrix in Figure 2.

Our *4d model* is derived from the *Kimura-2 model* of sequence evolution. The *4d alignment summary* is the vector (*x*,*y*,*s*,*g*) where *s* and *g* are as above, *x* is the number of *transversion mismatches* (between a purine and a pyrimidine or vice versa) and *y* is the number of *transition mismatches* (between purines or between pyrimidines). The four parameters are X, Y, S, and *G*. The matrix *w* of scores, as specified in [11], is now the middle matrix in Figure 2.

Our *5d scoring scheme* is derived from the *Kimura-3 model*. Here the matrix *w* is the rightmost matrix in Figure 2. The *5d alignment summary* is the vector (*x*,*y*,*z*,*s*,*g*), where *s* counts spaces, *g* counts gaps, *x* is the number of mismatches 
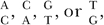
 *y* is the number of mismatches 
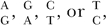
 and *z* is the number of mismatches 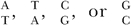. Thus, the 5d alignment summaries of the two *Drosophila* intron alignments at the beginning of the Introduction are (4,10,9,9,8) and (3,3,4,29,17). Even the 5d model does not encompass all scoring schemes that are used in practice. See the section “Assessment of the BLASTZ alignment” for a discussion of the BLASTZ scoring matrix [17] and its proximity to the Kimura-2 model.

Suppose we are given a specific alignment of two DNA sequences. Then the *robustness cone* of that alignment is the set of all parameter vectors that have the following property: any other alignment that has a different alignment summary is given a lower score. As a mathematical object, the robustness cone is an open convex polyhedral cone in the space *R^d^* of free parameters.

An alignment summary is said to be *optimal,* relative to one of our four models, if its robustness cone is not empty. Equivalently, an alignment summary is optimal if there exists a choice of parameters such that the Needleman–Wunsch algorithm produces *only* that alignment summary. Such a parameter choice will be robust, in the sense that if we make a small enough change in the parameters then the optimal alignment summary will remain unchanged. Each robustness cone is specified by a finite list of linear inequalities in the model parameters.

For example, consider the first alignment in the Introduction. Its 2d alignment summary is the pair (*x*,*s*) = (23,9), labeled **D** in Table 1. The robustness cone of this summary is the set of all points (*X*,*S*) such that the score 23*X* + 9*S* is larger than the score of all other alignments summaries other than (23,9). This cone is specified by the two linear inequalities *S* > *X* and 4*S* < 3*X*.

If we fix two DNA sequences, then the robustness cones of all the optimal alignments define a partition of the parameter space, *R^d^*. That partition is called the *alignment fan* of the two DNA sequences. Figure 1 shows the (biologically relevant part of the) alignment fan of two *Drosophila* introns in the 2d model. While this alignment fan has only 13 robustness cones, the alignment fan of the same introns has 76 cones for the 3d model, 932 cones for the 4d model, and 10,009 cones for the 5d model. These are the vertex numbers in Table 2.

**Table 2:**
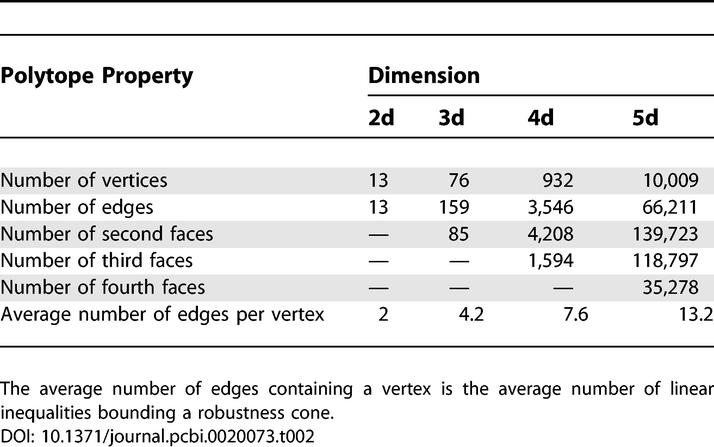
Face Numbers of the Alignment Polytopes for the Intron Sequences from the Beginning of the Introduction

#### Alignment polytopes.

The *convex hull* of a finite set *S* of points in *R^d^* is the smallest convex set containing these points. It is denoted conv(*S*) and called a *convex polytope*. There exists a unique smallest subset *V* ⊆ *S* for which conv(*S*) = conv(*V*). The points in *V* are called the *vertices* of the convex polytope. The vertices lie in higher-dimensional *faces* on the boundary of the polytope. Faces include *edges,* which are one-dimensional, and *facets,* which are (*d* − 1)-dimensional. Introductions to these concepts can be found in the textbooks [18,19]. By *computing the convex hull* of a finite set S ⊂ *R^d^,* we mean identifying the vertices and the facets of conv(*S*), and, if possible, all faces of all dimensions.

Consider one of the four models discussed in the previous section. The *alignment polytope* of two DNA sequences is the convex polytope conv(*S*) ⊂ *R^d^,* where *S* is the set of alignment summaries of all alignments of these two sequences. For instance, the 3d alignment polytope of two DNA sequences is the convex polytope in *R* ^3^ that is formed by taking the convex hull of all alignment summaries (*x*,*s*,*g*). Figure 3 shows the 3d alignment polytope for the two sequences in the Introduction. Its projection onto the (*x*,*s*)-plane is the polygon depicted in Figure 1.

**Figure 3:**
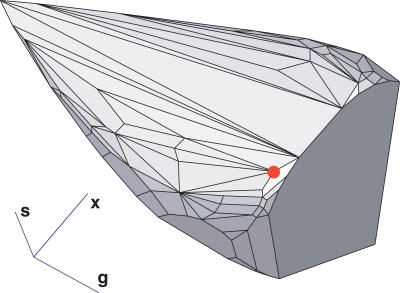
The Third Alignment Polytope of Our Two *Drosophila* Introns Has 76 Vertices The marked vertex (*x,s,g*) = (30,5,2) represents the BLASTZ alignment.

It is a basic fact of convexity that the maximum of a linear function over a polytope is attained at a vertex. Thus, an alignment of two DNA sequences is optimal if and only if its summary is in the set *V* of vertices of the alignment polytope. The Needleman–Wunsch algorithm efficiently solves the linear programming problem over this polytope. For instance, for the 3d model with fixed parameters, the alignment problem is the linear programming problem


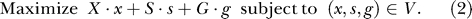


For a numerical example, consider the parameter values *X* = −200, *S* = −80, and *G* = −400, which represent an approximation of the BLASTZ scoring scheme (see the “Assessment of the BLASTZ alignment” section). The solution to Equation 2 is attained at the vertex (*x*,*s*,*g*) = (30,5,2), which is the 3d summary of the following alignment of our two *Drosophila* introns


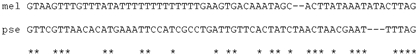


The 3d summary of this alignment is the marked vertex in Figure 3.

The problem of computing a parametric alignment is now specified precisely. The input consists of two DNA sequences. The output is the set of vertices and the set of facets of the alignment polytope conv(*S*). See also [11]. We note that the robustness cone of an optimal alignment summary is the *normal cone* of the polytope at that vertex. The alignment fan is the *normal fan* of the alignment polytope. See [11] for definitions of these concepts.

We considered two different methods for constructing all alignment polytopes for the two *Drosophila* genomes: *polytope propagation* and *incremental convex hull*. In our study we found that polytope propagation was outperformed by the incremental convex hull algorithm, especially for the higher dimensional models.

We now briefly outline the two methods. Polytope propagation for sequence alignment is the Needleman–Wunsch algorithm with the standard operations of plus and max replaced by Minkowski sum and polytope merge (convex hull of union). The polytope propagation algorithm was introduced in [10,11].

The incremental convex hull algorithm, on the other hand, gradually builds the alignment polytope by successively finding new optimal alignment summaries, the vertices of the polytope. To find the new optimal summaries, the algorithm repeatedly calls a *Needleman–Wunsch (NW) subroutine* that is an efficient implementation of the classical Needleman–Wunsch algorithm. For fixed values of the parameters, this subroutine returns an optimal alignment summary. For instance, for the 3d model, the input to the NW subroutine is a parameter vector (*X*,*S*,*G*) and the output is an optimal summary (*x*,*s*,*g*).

Suppose we have already found a few optimal alignment summaries, by running the NW subroutine with various parameter values. We let *P* be the convex hull of the summaries in *R^d^,* and we assume that *P* is already *d*-dimensional. We maintain a list of all vertices and facets of *P*. Each facet is either *tentative* or *confirmed,* where being confirmed means that its affine span is already known to be a facet-defining hyperplane of the final alignment polytope. In each iteration, we pick a tentative facet of *P* and an outer normal vector **U** of that facet. We then call the NW subroutine with **U** as the input parameter. The output of the NW subroutine is an optimal summary **v**. If the optimal score **U** · **v** equals the maximum of the linear function **U** · **w** over all **w** in *P,* then we declare the facet to be confirmed. Otherwise, the score **U** · **v** is greater than the maximum and we replace *P* by the convex hull of *P* and *v*. This convex hull computation utilizes the *beneath–beyond construction* [19], which erases some of the tentative facets of the old polytope and replaces them with new tentative facets.

The algorithm terminates when all facets are confirmed. The current polytope *P* at that iteration is the final alignment polytope. The number of iterations of this incremental convex hull algorithm equals the number of vertices plus the number of facets of the final polytope *P*. So for a given model, the running time of the incremental convex hull algorithm scales linearly in the size of the output. This was confirmed in practice by our computations (Table 3).

**Table 3:**
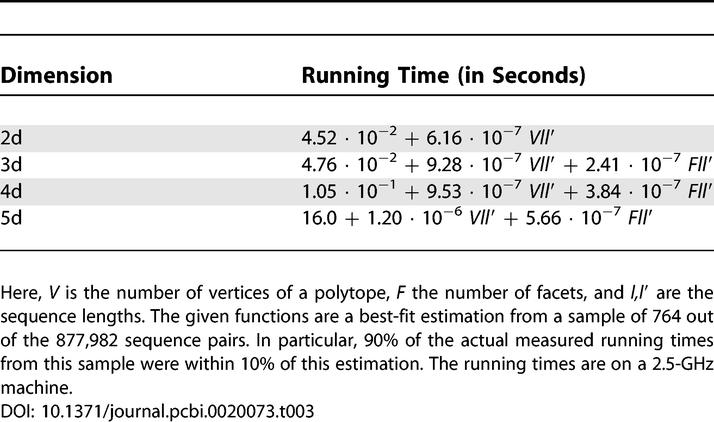
Observed Running Times of the Incremental Convex Hull Algorithm for Computing Alignment Polytopes

Our software, together with more details about our incremental convex hull implementation, is available for download at http://bio.math.berkeley.edu/parametric/.

Given an alignment polytope, there are various subsequent computations one may wish to perform. For instance, we may be interested in the robustness cones at the vertices. To get an irredundant inequality representation of a robustness cone, it suffices to know the edges emanating from the corresponding vertex. Thus it is useful to also compute the edge graph of each of our polytopes.

#### Computational results.

Using our implementation of the incremental convex hull algorithm described above, we computed the 2d, 3d, and 4d polytopes for each of the 877,982 segment pairs. We also computed 5d polytopes in many cases. These polytopes are available for downloading and viewing at http://bio.math.berkeley.edu/parametric/.

We empirically determined the expected CPU time to construct alignment polytopes. The results are reported in Table 3. As expected, the running time of the incremental convex hull algorithm scales linearly with the number of vertices plus facets. The running time of a single Needleman–Wunsch subroutine call scales linearly with the product *ll′* of the sequence lengths *l* and *l′*.

To effectively compute these polytopes, not only must we have an algorithm that runs quickly as a function of the number of vertices and facets, but also the number of vertices and facets must themselves be small. The theoretical bounds discussed in the Introduction ensure that these numbers grow polynomially, for any fixed *d*. In our computations we found that the numbers of vertices and facets of alignment polytopes are quite manageable even in dimensions 4 and 5. Averages of the numbers we actually observed are reported in Table 4.

**Table 4:**
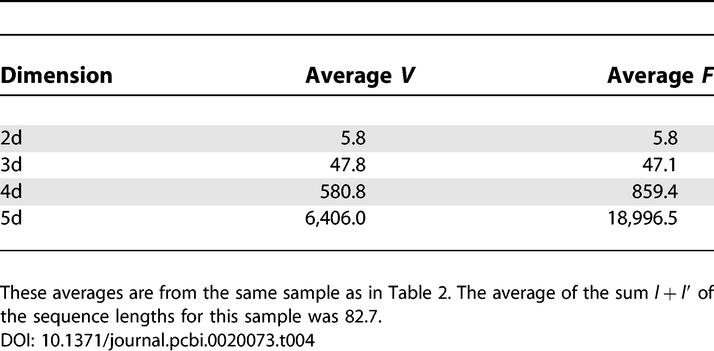
Averages of the Number *V* of Vertices and the Number *F* of Facets of Alignment Polytopes

We conclude our summary of computational results with a look at the alignment polytopes of the orthologous pair of introns in the Introduction. The sequence lengths are *l* = 60 and *l′* = 61. The 2d alignment polytope is shown in Figure 1. The 3d alignment polytope is shown in Figure 3. Table 2 reports statistics for the 2d, 3d, 4d, and 5d alignment polytopes for this sequence pair.

### From Polytopes to Biology

We describe three applications of our whole genome parametric alignment. First, we discuss how alignment polytopes are useful for parameter selection, and we assess the BLASTZ alignment of D. melanogaster and D. pseudoobscura. We then revisit the cis-regulatory element study in [20], and we determine alignments that identify previously missed conserved binding sites. Finally, we examine the problem of branch length estimation and provide a quantitative analysis of the dependence of branch length estimates on alignment parameters.

#### Assessment of the BLASTZ alignment.

A key problem in sequence alignment is to determine appropriate parameters for a scoring scheme. The standard approach is to select a model and then to identify parameter values that are effective in producing alignments that correctly align certain features. For example, the BLASTZ scoring matrix [17] was optimized for human–mouse alignment by finding parameters that were effective in aligning genes in the *HOXD* region. The BLASTZ scoring scheme is given by a scoring matrix called HOXD70 [17],


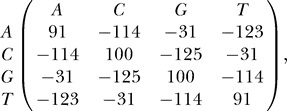


together with a space score of −30, and a gap score of −400. Although the HOXD70 matrix has six distinct entries, it can be approximated by a Kimura-2 matrix (Figure 2), since 91 is close to 100, and 114 is close to 123 and 125.

The alignment polytope of a pair of DNA sequences is a representation of all possible alignments organized according to a scoring scheme. Thus, our results and methodology make it possible, for the first time, to identify parameters that are guaranteed to optimize the alignment according to desired criteria. Moreover, our results offer biologists a mathematical tool for systematically assessing whether a proposed single alignment is suitable for its intended purpose.

We initiated such a study for the BLASTZ alignment [4] of D. melanogaster and *D. pseudoobscura,* which is available at http://genome.ucsc.edu. This alignment is widely used by biologists who study *Drosophila*. Although the BLASTZ alignment procedure is based on an initial “seeding” procedure (similar to our identification of constrained segment pairs), the alignments are then constructed using the Needleman–Wunsch algorithm with the HOXD70 matrix.

Recall that our orthology map consisted of 1,999,817 segment pairs: 1,116,792 consisted of segments for which we fixed the alignment (constrained segment pairs), and 883,025 were unconstrained segment pairs for which we constructed alignment polytopes. We found that the BLASTZ alignment agreed with 623,710 of our unconstrained segment pairs, of which 622,173 did not contain Ns. For each of these 622,173 segment pairs, we computed the 2d, 3d, and 4d alignment summaries for the BLASTZ alignments, and we determined whether or not they are optimal for some choice of model parameters.

We found that 269,186 (43.3%) of the BLASTZ alignments are vertices of the 3d polytope, but not the 2d polytope, and 201,982 (32.5%) are vertices of both the 2d and 3d polytopes. Only 151,004 (24.3%) of the BLASTZ alignments are not vertices of either the 2d or 3d alignment polytopes. In summary, our computations show that 32.5% of the BLASTZ alignments correspond to vertices of the 2d polytope and 75.7% correspond to vertices of the 3d polytope. These numbers are even higher for the 4d and 5d alignment polytopes.

Curiously, there is precisely one sequence pair where the BLASTZ alignment is a vertex of the 2d polytope but not the 3d polytope. This alignment is


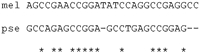


The 3d summary of this alignment is (11,3,2), which is the midpoint of the edge with vertices (11,3,1) and (11,3,3) on the 3d polytope. Hence this alignment is not optimal for any choice of parameters (*X*,*S*,*G*). However, it is optimal for the 2d model since the edge maps onto the vertex (11,3) of the 2d polygon.

Our results show that not only does the BLASTZ alignment agree well with our constrained segment pairs, but also the BLASTZ alignments are mostly vertices of the three dimensional polytopes, even on the unconstrained segment pairs. This suggests that there may be a statistical advantage to working with one of the lower dimensional models, and also indicates that the polytopes may be useful for finding parameters. We illustrate this point of view in the next section, where we identify vertices in the alignment polytope (and therefore parameter robustness cones) that are suitable for the alignment of cis-regulatory elements. Any user of the BLASTZ alignments may now use the alignment polytopes we provide to assess whether or not the fixed choice of the HOXD70 matrix is the right one for their particular biological application.

#### Conservation of cis-regulatory elements.

A central question in comparative genomics is the extent of conservation of cis-regulatory elements and the implications for genome function and evolution. Using our parametric alignment, we discovered that cis-regulatory elements may be more conserved between D. melanogaster and D. pseudoobscura than previously thought. Specifically, we used our alignment polytopes to examine the degree of conservation for 1,346 transcription factor binding sites [21] available at http://www.flyreg.org (we excluded 16 sites that were located in segment pairs containing *N*s). The 1,346 sites include the 142 sites examined by Richards et al. [20] in their comparison of D. pseudoobscura and D. melanogaster.

Specifically, for each of the 1,346 elements, we identified the orthologous segment pairs from our orthology map that contained the elements. We then extracted the polytopes from our whole genome parametric alignment. For each polytope, we determined an optimal alignment for which the number of matching bases of the corresponding element was maximized.

As an example, consider the transcription factor Adf1. It binds to a cis-regulatory element at chr3R:2,825,118–2,825,144 in D. melanogaster (Adf1-> Antp:06447 in the flyreg database). The BLASTZ alignment for this element is


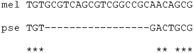


This alignment suggests that the D. melanogaster cis-regulatory element is not conserved in D. pseudoobscura. However, there are many optimal alignments that indicate that this element is conserved. Examining our constrained segment pairs, we found that the prefix TGTG was at the end of a 13-bp exact match. The remaining D. melanogaster element was part of a segment pair which has 813 distinct optimal alignments in the 3d model. Among these, we found the following alignment with parameters *G* = −3, *S* = −8, *X* = −18:


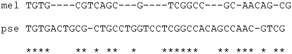


Note that we include the TGTG prefix to show a complete alignment of the cis-regulatory element. The second alignment has 24 matches instead of the BLASTZ alignment with eight. The number of matches can be used to calculate the *percent identity* for an element as follows:


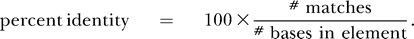


Percent identity was used in [20] as a criterion to determine whether binding sites are conserved. The BLASTZ alignment has 30% identity and the alignment with 24 matches has 89% identity. It is an optimal alignment with the highest possible percent identity. After examining all 813 optimal alignments, it appeared to us that the following alignment (obtained with *G* = −882, *S* = −87, *X* = −226) is more reasonable, even though it has a lower percent identity (67%):


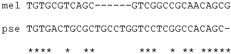


This alternative alignment suggests that the percent identity criterion may not be the best way to judge the conservation of elements. Regardless, we believe our parametric alignment indicates that in this particular case, the D. melanogaster cis-regulatory element is likely to have been conserved in D. pseudoobscura.

Our overall results are summarized in Table 5. We found that parameters can be chosen so as to significantly increase the number of matches for cis-regulatory elements. The “optimal parameters” row in the table shows results for the case where parameters were chosen separately for each segment pair so as to maximize the percent identity of the cis-regulatory elements. The “fixed parameters” row shows results when one parameter was selected (optimally) for all segment pairs simultaneously (this was only computed for the 2d model). Note that the mean per-site percent identity reported in [20] was 51.3%, considerably lower than what we found using the whole genome parametric alignment (even for the 2d model).

**Table 5:**
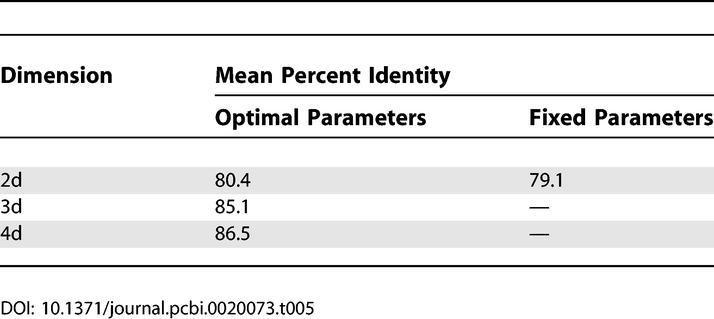
Cis-Regulatory Element Conservation

Our results seem to indicate that cis-regulatory elements are more conserved between D. melanogaster and D. pseudoobscura than previously thought. The alignment polytopes should be a useful tool for further investigation of the extent of conservation of cis-regulatory elements among the *Drosophila* genomes.

#### The Jukes–Cantor distance function.

An important problem in molecular evolution is the estimation of branch lengths from aligned genome sequences. A widely used method for estimating branch lengths is based on the Jukes–Cantor model of evolution [22]. Given an alignment of two sequences of lengths *l,l′,* with 2d alignment summary (*x*,*s*), one computes the *Jukes–Cantor distance* of the two genomes as follows:


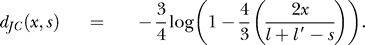


See [11] for a derivation of this expression, which is also known as the *Jukes–Cantor correction* of the two aligned sequences. The Jukes–Cantor distance can be interpreted as the expected number of mutations per site.

Because the Jukes–Cantor distance *d_JC_*(*x*,*s*) depends on the underlying pairwise sequence alignment summary, which in turn depends on the alignment parameters, it is natural to ask how the branch length estimate depends on the parameters in a 2d-scoring scheme. We therefore introduce the *Jukes–Cantor distance function* which is the function *JC*: *R^2^* → [0,∞) given by (*X*,*S*) 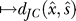 where 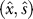 is the alignment summary maximizing *X* · *x* + *S* · *s*.

We computed the Jukes–Cantor distance function *JC* for the entire genomes of D. melanogaster and D. pseudoobscura. As the result of this computation, we now know the Jukes–Cantor distances for all whole genome alignments that are optimal for some choice of biologically reasonable parameters (*X*,*S*).

The notion of “optimal” used here rests on the following precise definitions. Given parameters (*X*,*S*), the optimal 2d alignment summary (*x*,*s*) for the two genomes is the sum of the optimal summaries of all 877,982 unconstrained segment pairs plus the sum of the alignment summaries of the non-coding constrained segment pairs (which do not depend on the parameters). We determined that the constrained segment pairs contained 91,355 mismatches and 16,339,305 matches. The *genome alignment polytope* is the Minkowski sum of the 877,982 alignment polytopes. The vertices of the genome alignment polytope correspond to optimal summaries of whole genome alignments.

We computed the genome alignment polytope for the 2d model. Remarkably, this convex polygon, which is the Minkowski sum of close to one million small polygons as in Figure 1, was found to have only 1,183 vertices. Moreover, of the 1,183 vertices of the genome alignment polytope, only 838 correspond to biologically reasonable parameters (*X* < 0, 2*S* < *X*). The finding that there are so few vertices constitutes a striking experimental validation of Elizalde's Few Inference Functions Theorem [11] in the context of real biological data.

The Jukes–Cantor distance function *JC* of D. melanogaster and D. pseudoobscura is a piecewise constant function on the (*X*,*S*)-plane. Indeed, *JC* is constant on the cones in the normal fan of the genome alignment polygon. Note that *JC* is undefined when (*X*,*S*) is perpendicular to one of the 1,183 edges of the genome alignment polygon. For such (*X,S)*, the Jukes–Cantor distance function jumps between its values on the two adjacent cones in the normal fan.

The graph of the Jukes–Cantor distance function is shown in Figure 4. The function ranges in value from 0.1253 to 0.2853, is monotonically decreasing as a function of *S,* and monotonically increasing as a function of *X*. We found it interesting that at the line *X = S,* there is a large “Jukes–Cantor jump” where the value of the function increases from 0.1683 to 0.2225.

**Figure 4:**
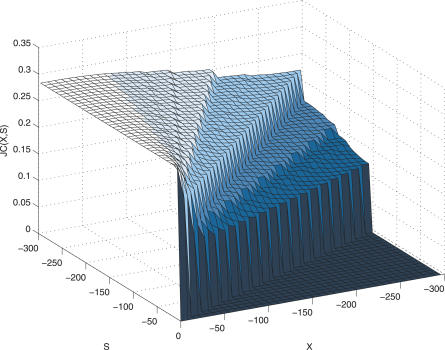
The Jukes–Cantor Distance Function of Two *Drosophila* Genomes

The Jukes–Cantor distance function is a new tool for parametric reconstruction of phylogenetic trees. Instead of estimating a single distance between each pair of genomes in a multiple species phylogenetic reconstruction, one can now evaluate the Jukes–Cantor function at vertices of the Minkowski sum of the whole genome alignment polytopes. These can be used for parametric phylogenetic reconstruction using distance-based methods such as neighbor joining.

## Discussion

The summary of a pair of aligned sequences is a list of numbers that determine the score for a scoring scheme. The alignment polytope is a geometric representation of the summaries of all alignments. It is an organizing tool for working with all alignments through their summaries. We view the Needleman–Wunsch algorithm as a fast subroutine for finding vertices of the alignment polytope. The construction of alignment polytopes is useful for biological studies based on sequence alignments where the conclusions depend on parameter choices.

We have highlighted three biological applications of our parametric alignments, namely the problem of parameter selection for sequence alignment, functional element conservation, and estimation of evolutionary rate parameters. In each case, our perspective suggests new directions for further research.

Alignment polytopes offer a systematic approach to solving the parameter selection problem. Although this paper did not address statistical aspects of parameter selection, we wish to emphasize that the vertices of the polytopes represent maximum a posteriori estimates of alignments for PHMMs. Our polytopes provide a setting for developing statistically sound methods for parameter selection that are not dependent on pre-existing alignments.

Our results on cis-regulatory elements show that they may be significantly more conserved than previously thought, and suggest that, in contrast to the analysis of ultra-conserved elements, sequence alignment procedures can be crucial in the analysis of certain functional elements. The ongoing *Drosophila* genome projects (consisting of sequencing 12 genomes of related species) offer an extraordinary opportunity for extending our study and further exploring cis-regulatory element conservation. This leads to the question of multiple alignment, which we have not addressed in this paper, but which we believe presents a formidable and important challenge in biological sequence analysis. In particular, it will be interesting to explore the geometric point of view we have proposed and to develop parametric algorithms for multiple sequence alignment.

The Jukes–Cantor distance function, computed here for the first time, will be important for determining the robustness of evolutionary studies based on sequence alignments. Estimates of the neutral rate of evolution, which are crucial for comparative genomics studies, can hopefully be improved and further developed using our mathematical tools. The Jukes–Cantor distance function opens up the possibility of parametric distance–based phylogenetic reconstruction. An immediate next step is the extension of our results to other phylogenetic models.

The construction of millions of alignment polytopes from two *Drosophila* genomes has revealed mathematical insights that should be explored further. For example, we observed empirically that alignment polytopes have few facets. Although we have not explored the combinatorial structure of alignment polytopes in this paper, this offers a promising direction for improving our parametric alignment algorithms and is an interesting direction for future research.

## Materials and Methods

The data analyzed are the genome sequences of D. melanogaster (April 2004 BDGP release 4) and D. pseudoobscura (August 2003 freeze 1).

Gene annotations for identifying exons were based on reference gene sets and *ab initio* predictions. For *D. melanogaster,* we used Flybase [2], SNAP, genscan, geneid, and RefSeq. Twinscan, SNAP, genscan, geneid, and xenoRefSeq (mRNAs from other species) were used for D. pseudoobscura. Annotations were obtained from the UCSC genome browser, except for SNAP which we ran ourselves.

MUMmer version 3.18 [23] was used to obtain potential non-coding anchors (20 bp exact matches). MUMmer was also run on orthologous segments determined by MERCATOR to identify ≥10 bp exact matches to refine the orthology map.

The Beneath–Beyond and polytope propagation algorithms were implemented in C++. Source code and binaries are available at http://bio.math.berkeley.edu/parametric/.

The BLASTZ alignment was obtained from the UCSC genome browser. The “net” and “chain” tracks were used to determine the best alignment for each interval in D. melanogaster. The resulting alignment blocks were compared with our constraints.

The transcription factor binding sites used in the cis-regulatory element study were obtained at http://www.flyreg.org. 16 sites were excluded because of segment pairs containing Ns.

Computations were carried out on an 18-node (36 CPU at 2.3 GhZ each) cluster. Each node had 2 Gb RAM.

The alignment polytopes, software, and supplementary material can be downloaded at http://bio.math.berkeley.edu/parametric/.

## Abbreviations

PHMM: pair hidden Markov model
